# Extended HPV genotyping by the BD Onclarity assay: concordance with screening HPV-DNA assays, triage biomarkers, and histopathology in women from the NTCC2 study

**DOI:** 10.1128/spectrum.00897-24

**Published:** 2024-11-22

**Authors:** Laura De Marco, Simonetta Bisanzi, Guglielmo Ronco, Pamela Mancuso, Francesca Carozzi, Elena Allia, Raffaella Rizzolo, Daniela Gustinucci, Helena Frayle, Jessica Viti, Anna Iossa, Elena Cesarini, Simonetta Bulletti, Basilio Passamonti, Silvia Gori, Laura Toniolo, Francesco Venturelli, Annarosa Del Mistro, Paolo Giorgi Rossi, Maria Benevolo, NTCC2 Working Group, Alessandra Barca

**Affiliations:** 1 Center for Cervical Cancer Screening, City of Health and Science Hospital, Turin, Italy; 2 Unit of Cancer Epidemiology and Center for Cancer Prevention (CPO), City of Health and Science Hospital, Turin, Italy; 3 Institute for Cancer Research, Prevention and Oncological Network (ISPRO), Florence, Italy; 4 Center for Cancer Epidemiology and Prevention (CPO), Turin, Italy; 5 Epidemiology Unit, Azienda Unità Sanitaria Locale—IRCCS di Reggio Emilia, Reggio Emilia, Italy; 6 Laboratorio Unico di Screening, USL Umbria 1, Perugia, Italy; 7 Istituto Oncologico Veneto IOV—IRCCS, Padua, Italy; 8 ULSS6 Euganea, Padua, Italy; 9 IRCCS—Regina Elena National Cancer Institute, Rome, Italy; National Chung Hsing University, Taichung, Taiwan

**Keywords:** human papillomavirus, cervical cancer, extended genotyping, NTCC2 study

## Abstract

The use of clinically validated human papillomavirus (HPV) assays is recommended in cervical cancer screening, and extended genotyping is getting attention as a triage biomarker because of the different oncogenic risk of the high-risk HPV genotypes. We compared the results of the Becton & Dickinson (BD) Onclarity HPV assay, on the residual baseline cervico-vaginal specimens of the NTCC2 trial, to those of the screening HPV-DNA assay (Cobas 4800 or HC2) and to cytology, p16/ki67 and E6/E7 mRNA triage results. We genotyped virtually all HPV-positive women and a consecutive sample of HPV-negatives. Among the 3,129 baseline-positives, 75.5% (*k* = 0.368) were BD-positive, as were 5 of the 333 baseline-negatives (1.5%). The concordance between BD and HPV-DNA screening test was 87% for Cobas (1,250/1,436) and 65.9% for HC2 (1,115/1,693). A higher than the recommended positivity threshold for Onclarity would increase the agreement but would not improve concordance in the overall screening population. Among the baseline-positive cases, we observed an increasing trend of BD positivity with cytology severity (from 71.6% in negative for intraepithelial lesion of malignancy to 95.1% in ASC-H+ samples), with histologically confirmed CIN3 (96.9%), with p16/ki67 dual staining positivity (90.9% among the positive and 69.6% among the negative specimens), and with E6/E7 mRNA positivity (93.4% in the mRNA-positive cases vs 39.7% among the mRNA-negatives). Our findings confirm some disagreement among different HPV assays used for screening. Nevertheless, the agreement is substantial for women with high-grade cytology, histologically confirmed CIN3, and p16/ki67 or mRNA positivity at triage, thus confirming a good clinical performance of all the tests used.

The NTCC2 trial is registered as Clinicaltrials.gov identifier NCT01837693.

**IMPORTANCE:**

Large randomized clinical trials have demonstrated that human papillomavirus (HPV) testing for high-risk types is more effective than cytology in detecting pre-cancerous lesions and preventing cervical cancer. Its use is being implemented in cervical cancer screening in several countries. The most recent guidelines recommend a risk-based management. It is therefore important to assess the individual risk of having/developing high-grade lesions of women testing high-risk HPV-positive. A crucial viral factor influencing the risk is the HPV genotype since different types are associated to different carcinogenetic risks. Understanding the degree of concordance among different assays targeting either HPV presence/type(s) or cellular morphology and proteins’ expression provides knowledge useful to better define how these tests can be used in screening protocols for an effective triage and to anticipate the possible implementation issues. Our study shows that the concordance between tests is higher when the infections have a higher probability of producing a clinically relevant lesion.

## INTRODUCTION

Cervical cancer is causally associated with persistent infection with high-risk human papillomavirus (hrHPV) types; the knowledge of the natural history of HPV infection and the long time needed for the development of invasive lesions has made its prevention a reality and its elimination a possibility (1). To reach this goal, integrated preventive and therapeutic measures need to be implemented for all women worldwide. HPV vaccination before 15 years of age (2) and population-based organized cervical screening constitute the basis for primary and secondary prevention. Screening for cervical cancer precursors with hrHPV molecular testing is now recommended by most scientific societies (3–5) and international agencies (6–8) upon demonstration, in comparison to cytology, of higher sensitivity, reproducibility, negative predictive value, and efficacy in preventing cervical cancer (9). The higher sensitivity is opposed to a lower specificity, which makes it necessary to include a triage strategy in case of hrHPV positivity, to discriminate women at higher risk of clinically significant precancerous lesions, who need immediate colposcopy, from women at lower risk that will undergo short-time retesting.

Cervical cancer screening guidelines recommend the use of clinically validated HPV assays (6, 10, 11). At present, less than 15 assays are clinically validated and recommended for use as primary tests in cervical cancer screening (12, 13). Current European protocols are mainly based on a positive or negative result for at least one hrHPV type, but since each of 12–14 hrHPV types causally related to cervical cancer differs in carcinogenicity (14), genotyping is getting attention as a triage biomarker. HPV16 ranks first (most carcinogenic) worldwide, and types 18 (frequently associated to adenocarcinoma), 31, 33, 35, 45, 52, and 58 bear a lower cancer risk than 16 but higher than other types, including HPV39, 51, 56, 59, and 68 (14). Among the clinically validated assays, some give only a pool result, and a few provide partial (for types 16 and 18) or extended (individual or by group) genotyping.

In Italy, the management of HPV-positive women is based on cumulative hrHPV positivity, but HPV genotyping has been performed and evaluated as a triage strategy in research studies (15–17).

The New Technologies for Cervical Cancer screening 2 (NTCC2) clinical trial is a multicentric randomized trial comparing mRNA-HPV testing (by Aptima, Hologic) and p16/ki67 expression (dual-stain, by CINTec PLUS, Roche) to liquid-based cytology as triage for HPV-DNA-positive women attending cervical cancer screening. In this trial, we made use of the clinically validated BD (Becton & Dickinson) Onclarity HPV assay (18–21) to perform an extended genotyping of the samples positive for HC2 or Cobas 4800. Onclarity is a real-time PCR-based test targeting the HPV E6 and E7 regions, identifying six types (HPV 16, 18, 31, 45, 51, and 52) individually and the others by grouping (22).

In the present study, we compared the results of the BD Onclarity HPV assay with extended genotyping on residual cervical specimens collected at baseline of the NTCC2 trial, to the results of the screening HPV-DNA assay (Cobas 4800 HPV or HC2) in all cases that resulted positive and in a sample of negative ones. We also compared the BD Onclarity genotyping results among samples that were positive for the original HPV DNA test stratified by the results of the other biomarkers tested in NTCC2, i.e., cytology, p16/ki67 dual staining, and E6/E7 mRNA. We aim to present data on the concordance between the different assays used in the NTCC2 study and on how the concordance changes in different groups with different biomarker’s status and different histology.

## MATERIALS AND METHODS

### Study population

Overall, 3,462 cervical cell samples collected in ThinPrep vials (Hologic) between April 2014 and February 2017, stored at −80°C for a median time of 5.5 years (range 4–7), were genotyped using the BD Onclarity HPV assay at the Center for Cervical Cancer Screening (Turin, Italy) and at the Institute for Cancer Research, Prevention and Oncological Network (ISPRO, Florence, Italy) laboratories. All samples from women who were HPV-positive at baseline were included in the study, as well as a series of HPV-negative ones collected from consecutive women during recruitment in Florence and Perugia (for sampling method, see [23, 24]).

Women were recruited at five Italian HPV DNA-based organized screening centers (23). High-risk HPV-DNA results were obtained by two different molecular methods: Hybrid Capture 2 (HC2, QIAGEN), which provides a pooled result of 13 hrHPV types, and Cobas 4800 HPV test (Roche), which detects 14 hrHPV types and provides a partial genotyping for HPV16 and 18 (24).

According to the NTCC2 study protocol, all the HPV DNA-positive women were also tested for two biomarkers: (i) E6/E7 mRNA by using Aptima HPV assay test (Hologic) that detects E6/E7 viral mRNA from 14 hrHPV types (16, 18, 31, 33, 35, 39, 45, 51, 52, 56, 58, 59, 66, and 68, detected as a pool) according to the manufacturer’s instructions; and (ii) p16/ki67 dual immunostaining, using the CINtec PLUS kit (Roche Diagnostics, Basel, Switzerland), according to the manufacturer’s instructions. Samples were scored as positive when double immunoreaction was revealed within at least one cell (23).

### BD Onclarity HPV assay

The BD Onclarity HPV assay is a real-time PCR that detects 14 hrHPV types and allows simultaneous individual identification of HPV 16, 18, 31, 45, 51, and 52. The remaining eight high-risk genotypes are brought together in three groups: P1 (HPV 33 and 58), P2 (HPV 56, 59, and 66), and P3 (HPV 35, 39, and 68). β-Globin gene is also simultaneously amplified in the same reaction mixes to check DNA quality from clinical samples and the assay performance. The assay was performed according to the manufacturer’s instructions by the BD Viper LT System on samples stored at −80°C for up to 7 years.

The presence or absence of clinically relevant HPV DNA is determined by the PCR cycle at which the signal crosses a pre-established threshold (cycle threshold [CT]). The manufacturer has set the positivity thresholds at CT 38.4 for HPV16 and at CT 34.2 for the other HPV types and the housekeeping (β-globin) gene; samples’ CT values are reported on the run report.

### Statistical analysis

The concordance analysis for all HPV-DNA results obtained by BD Onclarity and the HPV-DNA screening tests was performed by using a Cohens’ *k* test. A schematic summary of the hrHPV types detected by the three assays is reported in Table S1.

For the analysis, CT raw data for single-channel positivity and multiple infections that showed simultaneous positivity for two or more channels were also considered. The analyses were performed according to the positive cut-off values established by the manufacturer and by an arbitrary threshold (CT 40 for all channels, chosen because it is available for both real-time PCR assays) in order to extend the evaluation of concordance to the full array of results. A further comparison between HPV16 and HPV18 genotyping results by BD Onclarity and Cobas 4800 HPV assays was performed. The CT values for HPV16 and HPV18 of a consecutive series of samples collected in Veneto were also analyzed, comparing the CT values from BD Onclarity and Cobas 4800. The analyses of the CT values are explorative to see whether the optimization procedures made by the manufacturers are robust in the everyday practice on large screening populations.

We then compared the Cohen’s *k* values calculated for the study population samples with those that would be obtained in a screening population, i.e., weighting the HPV-negative women included in the study at baseline for their sampling fraction. Confidence intervals of the *k* values have been estimated using normal approximation.

The relationship between the *k* values and the level of agreement was based on Landis and Koch suggestion (25).

We also present sub-group analyses according to cytology, p16/ki67, E6/E7 mRNA, and histology results.

## RESULTS

**Fig 1:**
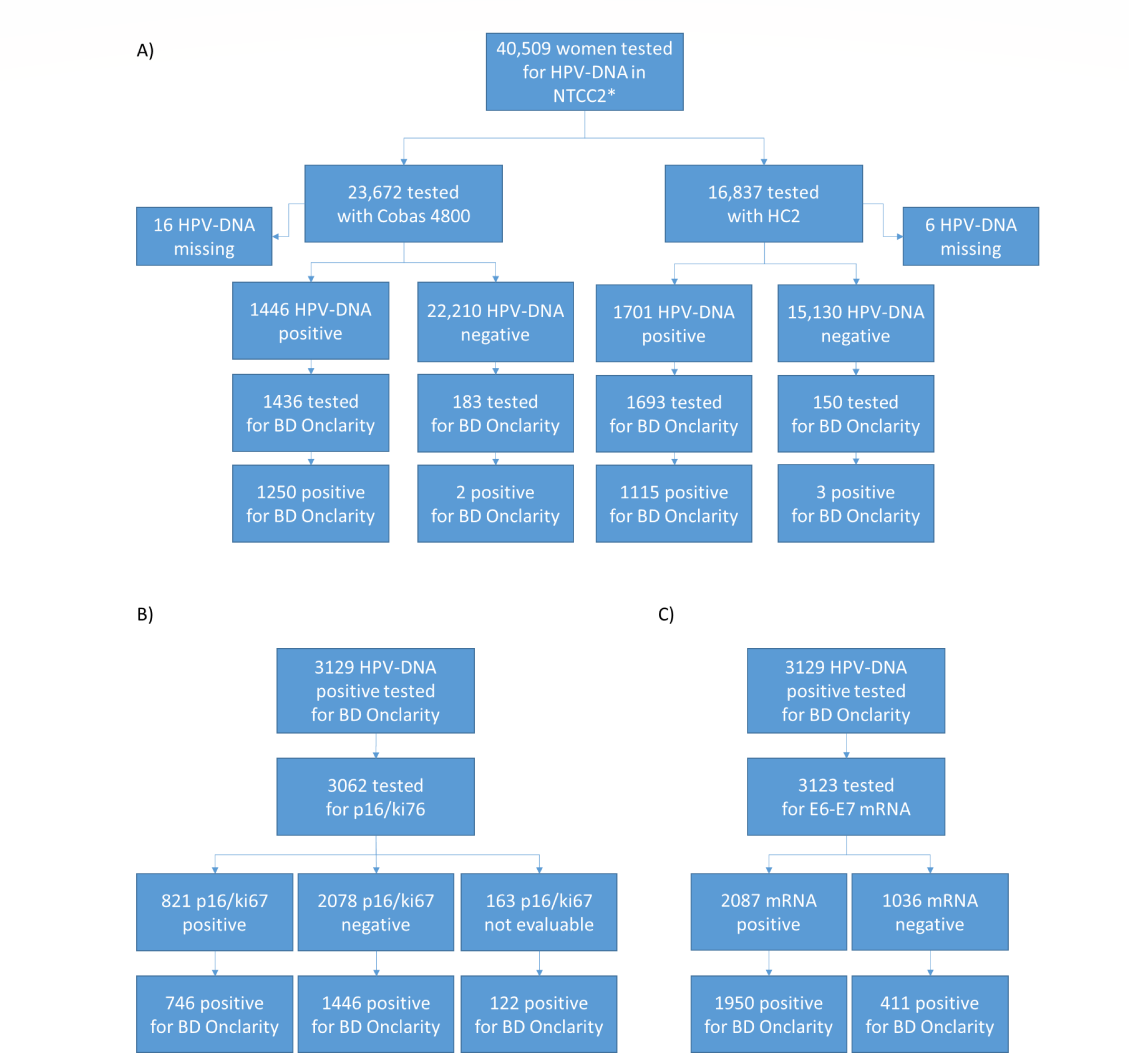
BD Onclarity test results performed on baseline samples of the NTCC2 study (**A**). BD Onclarity results on baseline HPV-positive samples according to p16/ki67 (**B**) and to E6-E7 mRNA results (**C**). *, This number does not include 618 women recruited in Trento for which there were no stored samples available for typing.

The NTCC2 study recruited 41,127 women, 40,509 of them in centers that stored samples for HPV typing (23,672 tested with Cobas 4800 HPV and 16,837 with HC2; Fig. 1). The HPV-positive samples were 3,147 (1,446 and 1,701, 6.1% and 10.1% with Cobas and HC2, respectively). In this study, almost all positive samples at baseline were retrieved for typing (1,436 tested by Cobas 4800 and 1,693 by HC2; Table 1). Furthermore, we retrieved 333 specimens HPV DNA-negative at baseline (183 tested by Cobas 4800 and 150 by HC2; Fig. 1). Among the 3,462 baseline samples tested with the BD Onclarity assay, an invalid result was obtained for three samples (two positives by Cobas 4800 and one by HC2 test). Two thousand three hundred seventy samples were HPV DNA-positive for at least one genotype (68.5%), and among them, 500 showed simultaneous positivity for two or more channels.

**TABLE 1:** BD Onclarity typing test according to baseline HPV DNA assay on baseline samples of Cobas/HC2 HPV DNA-positive women

Type	Cobas any positivity		Cobas						HC2	
			HPV16		HPV18		Other HR			
BD Onclarity type	*n*	%	*n*	%	*n*	%	*n*	%	*n*	%
Samples analyzed	1,436		290^*a*^		106^*a*^		1,175^*a*^		1,693	
16	291	20.3	276	95.2	12	11.3	105	8.9	241	14.2
18	91	6.3	10	3.4	83	78.3	35	3.0	60	3.5
45	61	4.2	3	1.0	4	3.8	61	5.2	62	3.7
33/58	173	12.0	20	6.9	0	0.0	170	14.5	144	8.5
31	227	15.8	22	7.6	9	8.5	226	19.2	187	11.0
56/59/66	340	23.7	34	11.7	14	13.2	337	28.7	277	16.4
51	113	7.9	8	2.8	3	2.8	113	9.6	107	6.3
52	128	8.9	14	4.8	7	6.6	124	10.6	109	6.4
35/39/68	177	12.3	20	6.9	11	10.4	174	14.8	194	11.5
Negative	186	13.0	10	3.4	15	14.2	163	13.9	578	34.1
At least one channel positives	1250	87.0	280	96.6	91	85.8	1,012	86.1	1,115	65.9
Multichannel positives	278	19.4	95	32.8	35	33.0	260	22.1	221	13.1

^*a*^ One hundred twenty-nine positive results were positive for more than one Cobas channel.

Overall, the BD Onclarity positivity among the 3,129 baseline HPV-positive samples by Cobas/HC2 reached 75.5%. Five out of the 333 baseline (two Cobas and three HC2) HPV-negative samples showed HPV positivity with the BD assay (1.5%). The overall concordance in the study group was 77.8% (for a Cohen’s *k* of 0.37; Table 2), but applying the observed positivity rates (according to the weighted calculation of the fraction of HPV-negative women included) to a screening population, where the HPV-negatives are the vast majority, the estimated concordance would be 96.7% for a *k* of 0.76. The agreement was higher with Cobas HPV DNA test (88.4%; *k* in the study population = 0.60; *k* for the screening population = 0.84) than with HC2 (68.5%; *k* in the study population = 0.23; *k* for the screening population = 0.69; Table 2). In addition, we restricted the comparison between BD Onclarity and the other HPV-DNA assays, to the consecutive samples from Florence and Perugia from which the HPV-negative specimens were drawn, representing an unbiased and complete set of samples. The findings from these sensitivity analyses confirmed those of the unrestricted analyses (Tables S2 and S3).

**TABLE 2:** Agreement between results of the original HPV-DNA test and of BD Onclarity in the study population and in a screening population by using the manufacturer’s CT value cut-off

		Any original HPV test			
		Positive	Negative	Total	Agreement in the sample 77.8%
BD Onclarity	Positive	2,365	5	2,370	Cohen kappa in the sample 0.37 (95% CI 0.33–0.41)
	Negative	764	328	1,092	Agreement in the screening population^*a*^ 96.7%
	Total	3,129	333	3,462	Cohen kappa in screening pop. ^*a*^ 0.76 (95% CI 0.75–0.78)

		HC2			
		Positive	Negative	Total	Agreement in the sample 68.5%
BD Onclarity	Positive	1,115	3	1,118	Cohen kappa in the sample 0.23 (95% CI 0.18–0.29)
	Negative	578	147	725	Agreement in the screening population^*a*^ 94.8%
	Total	1,693	150	1,843	Cohen kappa in screening pop. ^*a*^ 0.69 (95% CI 0.67–0.71)

		Cobas 4800			
		Positive	Negative	Total	Agreement in the sample 88.4%
BD Onclarity	Positive	1,250	2	1,252	Cohen kappa in the sample 0.60 (95% CI 0.54–0.65)
	Negative	186	181	367	Agreement in the screening population^*a*^ 98.2%
	Total	1,436	183	1,619	Cohen kappa in screening pop. ^*a*^ 0.84 (95% CI 0.83–0.86)

HPV16		Cobas 4800			
		Positive	Negative	Total	Agreement in the sample 98.0%
BD Onclarity	Positive	276	15	291	Cohen kappa in the sample 0.94 (95% CI 0.91–0.96)
	Negative	14	1,131	1,145	Agreement in the screening population^*a*^ 98.0%
	Total	290	1,146	1,436	Cohen kappa in screening pop. ^*a*^ 0.94 (95% CI 0.91–0.96)

HPV16/HPV18		Cobas 4800			
		Positive	Negative	Total	Agreement in the sample 96.4%
BD Onclarity	Positive	353	19	372	Cohen kappa in the sample 0.91 (95% CI 0.88–0.93)
	Negative	32	1,032	1,064	Agreement in the screening population^*a*^ 96.5%
	Total	385	1,051	1,436	Cohen kappa in screening pop. ^*a*^ 0.91 (95% CI 0.88–0.93)

^*a*^ The baseline samples negative for the original HPV test are weighted to estimate the agreement and kappa values in a screening population with overall HPV DNA positivity of 7.8% (6.1% for Cobas 4800 and 10.1% for HC2).

### Typing results

**Fig 2:**
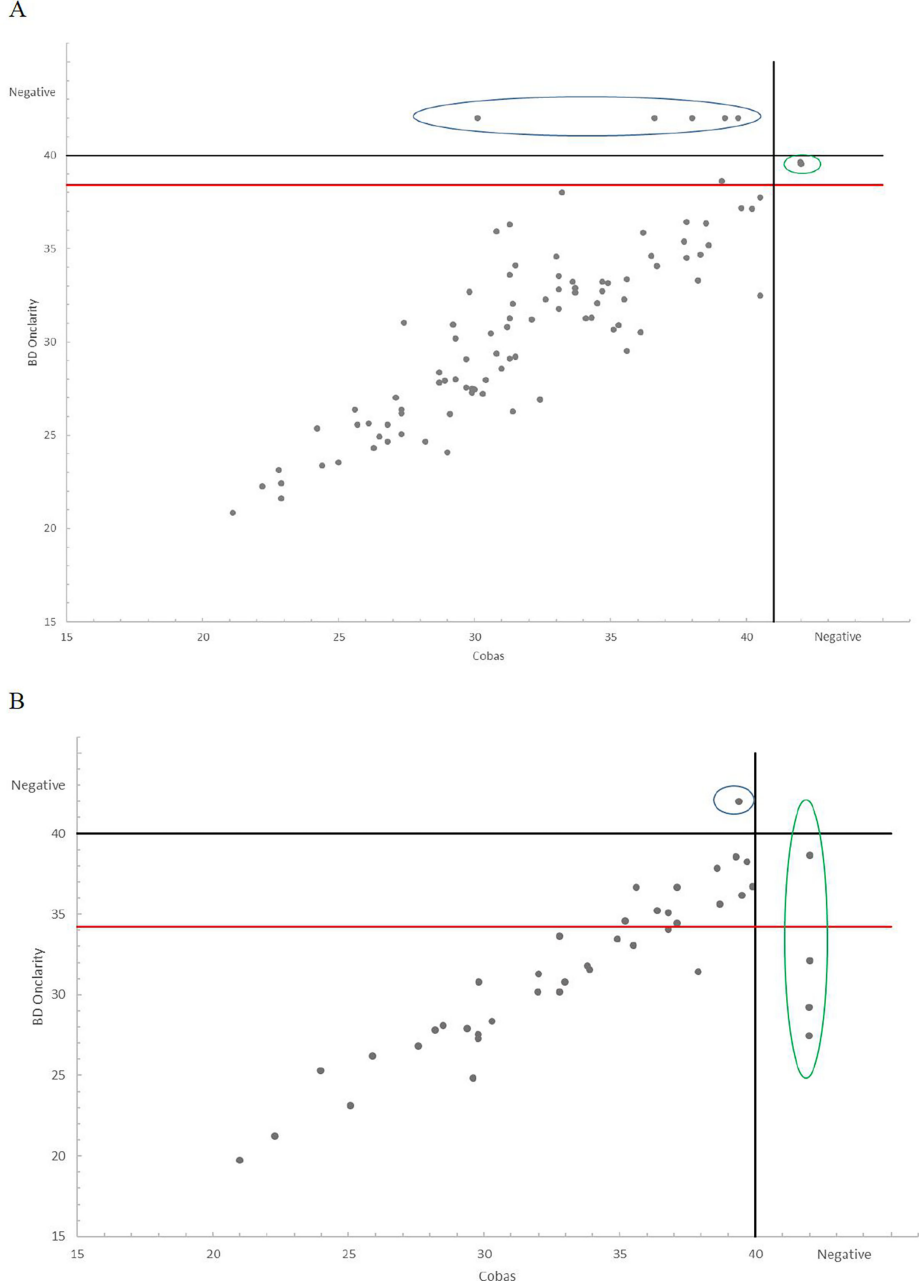
Comparison of CT values between BD Onclarity and Cobas 4800. (A) Analysis of 95 samples positive for HPV16. The black vertical line represents the Cobas 4800 cut-off value (40.5 for HPV16). Red line represents the CT value (38.4) for HPV16 positivity defined by the manufacturer. Only three samples showed CT values above the cut-off. The samples (*N* = 5) grouped in the blue circle represent samples negative for BD Onclarity but positive for Cobas. Samples (*N* = 2) grouped in the green circle represent BD Onclarity-positive but Cobas-negative samples. (B) Analysis of 42 samples positive for HPV18. The black vertical line represents the Cobas 4800 cut-off value (40 for HPV18). Red line represents the CT value (34.2) for HPV18 positivity defined by the manufacturer. Thirteen samples showed CT values above the cut-off. The samples (*N* = 1) grouped in the blue circle represent samples negative for BD Onclarity but positive for Cobas. Samples (*N* = 4) grouped in the green circle represent BD Onclarity-positive but Cobas-negative samples.

Among the samples positive on Cobas or HC2, HPV 16 was recorded in 532 (17%), HPV18 in 151 (4.8%), and HPV31 in 414 (13.2%), while positivity for the P2 channel (detecting HPV types 56, 59, 66) was recorded in 617 (19.7%) specimens. The Cobas assay provides partial genotyping for HPV16 and HPV18 and a pooled non16/18 HR types (“other HR”), and we found that the concordance with BD Onclarity was 95.2% and 78.3% for HPV16 and HPV18, respectively (Table 1). In a consecutive series of specimens collected in Veneto, we compared the CT values from BD Onclarity and from Cobas 4800, for HPV16 and for HPV18. Discordant results were more frequent for HPV18 (13/42, 30%) than for HPV16 (5/95, 3%). The analyses of the CT values for these samples are shown in Fig. 2, A and B; considering also CT values higher than the cut-off (up to 40), a linear correlation was observed for most samples, with non-linearity for only very few samples.

In the five samples that were positive for BD Onclarity and negative for baseline HPV test, four single and one double positivity, we revealed two HPV16 (0.6%), one HPV45 (0.3%), and three P2 (56, 59, 66) pooled genotypes (0.9%).

### Effect of threshold

**Fig 3:**
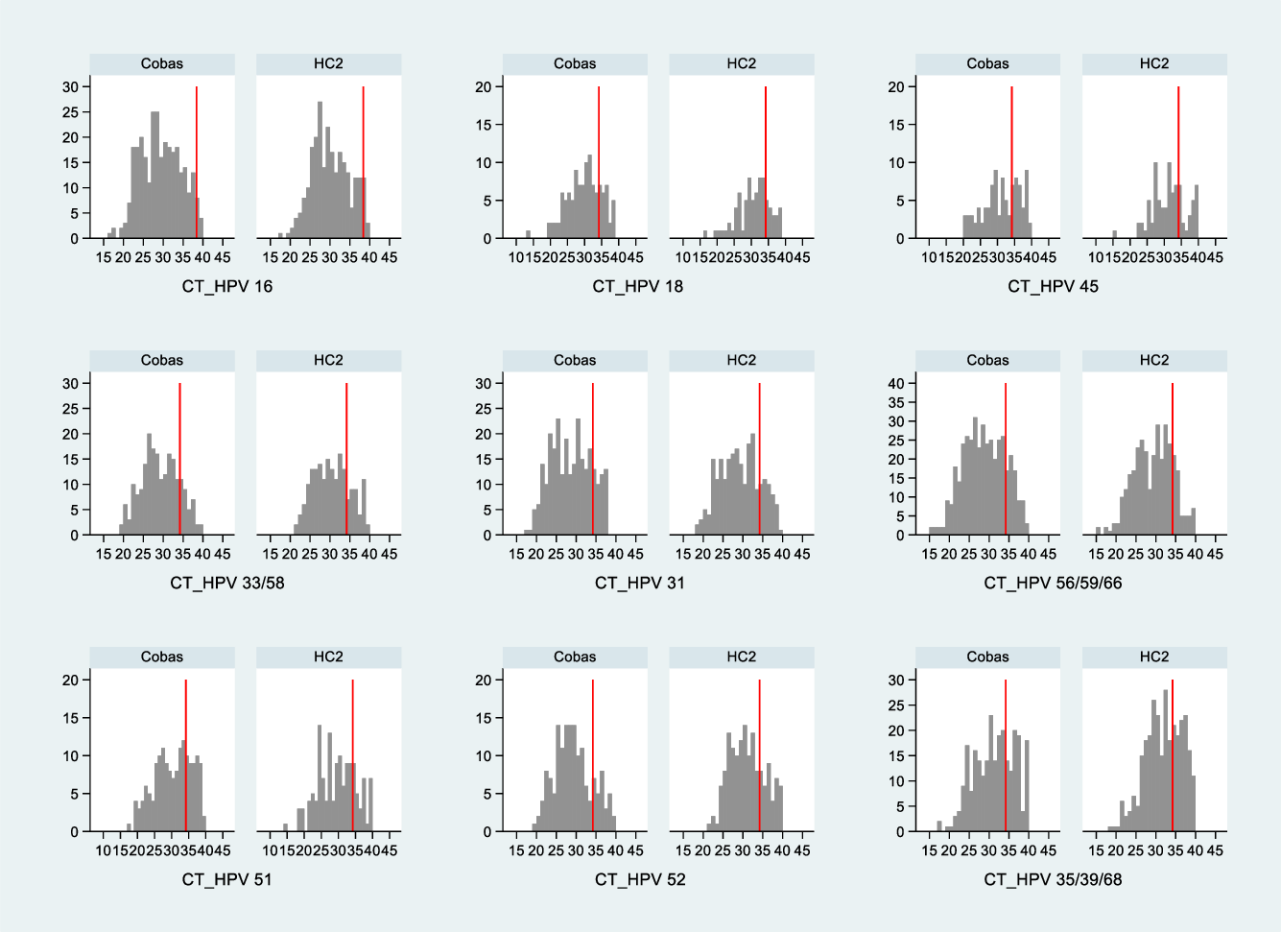
Quantitative results by HPV-DNA assay and BD Onclarity PCR channel on baseline samples of Cobas/HC2 HPV-DNA-positive women stratified by HPV type. The vertical red line indicates the manufacturer-assessed CT value for positivity: CT 38.4 for HPV16 and CT 34.2 for the housekeeping gene and/or the other HPV types.

Figure 3 shows the quantitative distribution of the BD Onclarity results in terms of CT values for each channel, stratified by HPV DNA test (HC2 or Cobas) that determined the positivity at baseline. Raw data for single-channel positivity, as well as multiple infections, were considered for the analysis. No clear pattern in the distribution of BD Onclarity CT values was identified, with some channels showing lower CT values in samples that were tested with Cobas and other channels showing lower CT values in samples tested with HC2.

If the raw CT values over the positivity threshold set by the manufacturer (i.e., negative) were considered, 332 of the 764 samples that were positive at the baseline HPV test but negative by BD Onclarity test showed detectable amplification within the 40th cycle of PCR amplification (data not shown). At the threshold of the 40th cycle of amplification, the agreement in the study population would be higher than the values observed at manufacturer’s thresholds (overall 87.0%, for a kappa of 0.52), but agreement and kappa values would decrease in the screening population (overall 95.0%, for a kappa of 0.70; Table 3). It is worth noting that only eight samples showed amplification in the HPV16 channel, while most of the samples (*N* = 77) showed amplification in group P3 (HPV 35, 39, and 68). The results were similar when restricting the analyses to Perugia and Florence.

**TABLE 3:** Agreement between results of the original HPV-DNA test and of BD Onclarity at the threshold of ≤40 CT for all channels (raw data) in the study population and in a screening population by using a modified CT cut-off

		Any original HPV test			
		Positive	Negative	Total	Agreement in the sample 87.0%
BD Onclarity	Positive	2,692	14	2,706	Cohen kappa in the sample 0.52 (95% CI 0.48–0.56)
	Negative	437	319	756	Agreement in the screening population 95.0%
	Total	3,129	333	3,462	Cohen kappa in screening pop. 0.70 (95% CI 0.69–0.71)

		HC2			
		Positive	Negative	Total	Agreement in the sample 77.4%
BD Onclarity	Positive	1,286	9	1,295	Cohen kappa in the sample 0.32 (95% CI 0.26–0.37)
	Negative	407	141	548	Agreement in the screening population 92.1%
	Total	1,693	150	1,843	Cohen kappa in screening pop. 0.59 (95% CI 0.57–0.61)

		Cobas 4800			
		Positive	Negative	Total	Agreement in the sample 97.8%
BD Onclarity	Positive	1,406	5	1,411	Cohen kappa in the sample 0.90 (95% CI 0.86–0.93)
	Negative	30	178	208	Agreement in the screening population 97.3%
	Total	1,436	183	1,619	Cohen kappa in screening pop. 0.80 (95% CI 0.79–0.82)

HPV16		Cobas 4800			
		Positive	Negative	Total	Agreement in the sample 98.0%
BD Onclarity	Positive	278	17	295	Cohen kappa in the sample 0.94 (95% CI 0.92–0.96)
	Negative	12	1,129	1,141	Agreement in the screening population^*a*^ 98.0%
	Total	290	1,146	1,436	Cohen kappa in screening pop. ^*a*^ 0.94 (95% CI 0.92–0.96)

HPV16/HPV18		Cobas 4800			
		Positive	Negative	Total	Agreement in the sample 97.4%
BD Onclarity	Positive	369	21	390	Cohen kappa in the sample 0.93 (95% CI 0.91–0.96)
	Negative	16	1,030	1,046	Agreement in the screening population^*a*^ 97.4%
	Total	385	1,051	1,436	Cohen kappa in screening pop. ^*a*^ 0.93 (95% CI 0.91–0.96)

^*a*^ The baseline samples negative for original HPV test are weighted to estimate the agreement and kappa values in a screening population with overall HPV DNA positivity of 7.8% (6.1% for Cobas 4800 and 10.1% for HC2).

### BD Onclarity results according to other biomarkers results

BD Onclarity-positive results were stratified according to cytology reports of the baseline Cobas/HC2 HPV-positive samples. An overall trend of increasing BD Onclarity positivity was observed from negative for intraepithelial lesion or malignancy (NILM; 71.6%) to ASC-H+ cytological samples (95.1%; Table 4). It is worth noting that positivity for HPV16 also increased from 13.9% in NILM to 47.8% in ASC-H+ samples, and HPV18 and HPV33/58 showed the same trend albeit to a lesser extent. However, HPV45, HPV31, HPV35/39/68, HPV51, HPV52, and HPV56/59/66 showed the highest positivity rate in the ASC-US/L-SIL category (Table 4). Noteworthy, among the 751 Cobas or HC2 HPV-positive samples testing negative with the BD assay, the percentage was the highest among NILM (651, 28.4%) and the lowest among ASC-H+ samples (9, 4.9%; Table 4).

**TABLE 4:** BD Onclarity typing result distribution according to Cobas/HC2 HPV DNA and cytology results on baseline samples^*a*^

BD Onclarity channel	Cobas/HC2 HPV DNA+							
	NILM		ASC-US/L-SIL		ASC-H+		Inadequate or missing	
	*n*	%	*n*	%	*N*	%	*n*	%
Samples	2,295		606		184		44	
16	319	13.9	115	19.0	88	47.8	10	22.7
18	105	4.6	34	5.6	11	6.0	1	2.3
45	89	3.9	30	5.0	3	1.6	1	2.3
33/58	212	9.2	72	11.9	27	14.7	6	13.6
31	284	12.4	98	16.2	28	15.2	4	9.1
56/59/66	420	18.3	173	28.5	18	9.8	6	13.6
51	138	6.0	65	10.7	13	7.1	4	9.1
52	165	7.2	57	9.4	14	7.6	1	2.3
35/39/68	270	11.8	82	13.5	16	8.7	3	6.8
Negative	651	28.4	91	15.0	9	4.9	13	29.5
At least one channel positives	1,644	71.6	515	85.0	175	95.1	31	70.5
Multichannel positives	294	12.8	163	26.9	37	20.1	5	11.4

^*a*^ NILM, negative for intraepithelial lesion or malignancy; ASC-US, atypical squamous cells of undetermined significance; L-SIL, low-grade squamous intraepithelial lesion; ASC-H, atypical squamous cells—cannot exclude high-grade squamous intraepithelial lesion.

Overall, 3,062 Cobas or HC2 HPV DNA-positive samples were tested by BD Onclarity assay and p16/ki67; 746 of 821 (90.9%) p16/ki67-positive samples were BD Onclarity-positive for at least one genotype, and 246 (30%) were HPV16-positive (Table 5). Overall, among the p16/ki67-negative samples, the BD Onclarity positivity for at least one genotype was 69.6% and 11.8% for HPV 16. The positivity rates of all genotypes, except the P2 group (HPV 56, 59, and 66) and HPV51, were lower in the p16/ki67-negative samples than those observed in the p16/ki67-positive samples.

**TABLE 5:** BD Onclarity result distribution according to p16/ki67 results on baseline Cobas/HC2 HPV DNA-positive samples

BD Onclarity type	p16/ki67+		p16/ki67−		Not evaluable	
	*n*	%	*N*	%	*n*	%
Samples analyzed^*a*^	821		2,078		163	
16	246	30.0	246	11.8	25	15.3
18	51	6.2	92	4.4	2	1.2
45	35	4.3	79	3.8	8	4.9
33/58	121	14.7	170	8.2	22	13.5
31	144	17.5	241	11.6	20	12.3
56/59/66	149	18.1	423	20.4	32	19.6
51	54	6.6	159	7.7	5	3.1
52	82	10.0	137	6.6	9	5.5
35/39/68	115	14.0	221	10.6	27	16.6
Negative	75	9.1	632	30.4	41	25.2
At least one channel positives	746	90.9	1,446	69.6	122	74.8
Multichannel positives	202	24.6	261	12.6	24	14.7

^*a*^ Only samples with a p16/ki67 test performed were included (*n* = 3,062).

BD genotyping results were also analyzed according to the HPV E6/E7 mRNA results on the 3,123 Cobas/HC2-positive samples with a valid mRNA result (Table 6). The positivity rate for every channel was higher in mRNA-positive compared to mRNA-negative samples. Among the 2,087 mRNA-positive samples, 1,950 (93.4%) showed at least one BD-positive channel, and 473 of them (22.7%) showed more than one positive channel. In contrast, positivity among the mRNA-negative samples was observed in only 411 samples (39.7%), of which 25 (2.4%) for more than one channel (Table 6).

**TABLE 6:** BD Onclarity result distribution according to E6/E7 mRNA results on baseline Cobas or HC2 HPV DNA-positive samples

BD Onclarity type	mRNA+		mRNA−	
	*n*	%	*n*	%
Samples analyzed^*a*^	2,087		1,036	
16	450	21.6	82	7.9
18	128	6.1	23	2.2
45	101	4.8	22	2.1
33/58	296	14.2	20	1.9
31	373	17.9	40	3.9
56/59/66	502	24.1	115	11.1
51	166	8.0	52	5.0
52	199	9.5	37	3.6
35/39/68	326	15.6	45	4.3
Negative	137	6.6	625	60.3
At least one channel positives	1,950	93.4	411	39.7
Multichannel positives	473	22.7	25	2.4

^*a*^ Only samples with a valid E6/E7 mRNA test performed were included (*n* = 3,123).

The quantitative distribution of the BD results in terms of CT values for each channel stratified according to the results of these biomarkers showed lower CT values among positive than negative samples, as shown in Fig. S1 for cytology (considering ASC-US or more severe report as the threshold), in Fig. S2 for p16/ki67 and in Fig. S3 for E6/E7 mRNA results. Moreover, also the proportion of CT values over the negativity threshold was much lower among samples negative for the biomarkers.

### BD Onclarity results in high-grade lesions

Overall, we found 174 CIN2+, of which 96 CIN3. Based on the BD Onclarity genotyping results (Table 7), among the 174 CIN2+, 168 (96.5%) showed at least one BD-positive channel (multi-channel positivity in 24%), of which 82 (48.8%) were HPV16 infected, while among the CIN3 96.9% (93/96) showed at least one positive channel, of which 52 (54.2%) were positive for HPV16. Compared to multi-channel positive women, those positive for a single channel had a higher CIN3/CIN2 ratio (1.4 vs 1), while no significant differences emerged by age at enrolment (grouped as <35, 35–50, and >50 years; Table S4).

**TABLE 7:** Clinical outcome (with CIN2 and CIN3 histologically confirmed) in Cobas/HC2 HPV DNA-positive women, according to BD Onclarity result distribution on baseline samples

Type	No. of women with complete follow-up^*a*^	No CIN^*b*^		CIN2		CIN3	
		*N*	%	*N*	%	*N*	%
Samples analyzed	2,663	2,489		78		96	
16	448	366	14.7	30	38.5	52	54.2
18	131	124	5	4	5	4	4
45	111	105	4.2	3	3.8	3	3
33/58	275	251	10.1	11	14	13	13.5
31	346	312	12.5	22	28	12	12.5
56/59/66	525	500	20.1	11	14	14	14.6
51	192	183	7.4	4	5	5	5.2
52	197	187	7.5	4	5	6	6.2
35/39/68	326	303	12.2	14	18	9	9.4
Negative	647	641	25.8	3	3.8	3	3
At least one channel positives	2,016	1,848		75		93	
Multichannel positives	433	392		23	30.7	18	19.4
Single-channel positives	1,583	1,456		52	69.3	75	80.6

^*a*^ Four hundred sixty-six women had no complete follow-up at 24 months since recruitment, i.e., had a positive HPV as last available result without any colposcopy. Complete follow-up includes women who tested negative for HPV or women with colposcopy and, when required, colposcopy-guided biopsy.

^*b*^ Includes 492 women with HPV-negative test at follow-up, women with negative colposcopy and no biopsy (1,095) and women with negative biopsy or CIN1 (902) CIN: cervical intraepithelial neoplasia.

## DISCUSSION

In this study, we compared the results of the BD Onclarity assay on the residual samples collected at baseline of the NTCC2 trial, to the results of the screening HPV-DNA assay and of the other triage biomarkers (cytology, p16/ki67, and E6/E7 mRNA), as well as to histology.

We observed a sizable difference in the rate of samples that resulted concordantly positive by HC2 and Cobas vs BD Onclarity; namely, 68.5% and 88.4%, respectively, with a fair (*κ* values ranged from 0.23 to 0.60) concordance. This concordance improves (*κ* = 0.52) when the raw data (including those above threshold) are considered; nevertheless, such a lowering of the positivity threshold would negatively impact specificity with a large increase in BD Onclarity-positives among the Cobas or HC2-negatives. HC2 and Cobas HPV assays differ in methodology (full-genome hybridization and signal amplification for HC2, L1-based real-time PCR for Cobas 4800) and performance (26). Previous publications have highlighted a disagreement on the result when different HPV assays were compared, referring to several factors, including chemistry, amplicon size, and targeted HPV types (26–28). The genotyping assay used in this study is a real-time PCR, a methodology much more similar to Cobas 4800 than to HC2; this may explain the higher agreement between Cobas and BD Onclarity than between HC2 and BD Onclarity. Another likely explanation for discordance between HPV assays is the occurrence of cross-reactivity with low-risk genotypes due to sequence homology or non-specific amplifications. Cross-reactivity has been most frequently observed for HC2 (29) and less frequently for Cobas 4800 (30); the most frequently cross-reacting types are 53, 61, 62, 70, and 82. HC2 showed a cross-reactivity also for HPV66, but this could rather increase agreement with other tests since this genotype is among the ones targeted by BD Onclarity and Cobas 4800.

In our study, samples were collected between 2014 and 2017 (and immediately tested by HC2 or Cobas 4800) and analyzed by BD Onclarity in 2021; amplification of the housekeeping gene was recorded in all but three (out of 3,462) samples, and samples stored for >5 years had an overall BD positivity rate higher than samples stored for <5 years (Table S5), indicating that valid results can be obtained on PreservCyt specimens stored at −80°C for up to 7 years (a much longer time than the 180 days indicated for storing at −20°C in the instructions for use). The use of frozen samples is in line with the VALGENT (Validation of HPV Genotyping Tests) protocols used for clinical validation of HPV assays for cervical screening; in the study by Polman et al. (31), the VALGENT-3 samples were tested by the HPV-Risk assay (in comparison to the results of the HC2 test performed at the time of collection) after storage at −70°C for up to 6 years. Moreover, the Cobas/HC2-positive/Onclarity-negative proportion is higher in all the biomarker profiles linked to lower risk of immediate or future CIN3. Nevertheless, even if the trend suggests that the overall population had stable target based on the correlation provided, individual specimen issues could not be ruled out.

Among the HPV types individually detected by BD, HPV16 and HPV31 were the most frequent, while the P2 group (HPVs 56/59/66) showed the highest prevalence. There are few population-based studies on the prevalence of HPV types in Italy, probably due to the use of assays with a partial genotyping instead of a complete genotyping in organized screening. A recent study (32) evaluated the prevalence of HPV types in the metropolitan area of Naples finding a high proportion of HPV16 (14.6%) followed by HPV31 (13.8%), consistent with our findings. In Canada, in a concordance study between BD Onclarity and HC2-positive samples, Volesky et al. (33) described a high prevalence of P2 (56/59/66) HPVs, followed by HPV16, P3 (35/39/68) HPVs, and then HPV31.

Unfortunately, the number of negative samples tested to assess specificity was small; thus, our estimate of the proportion of BD Onclarity positivity in women negative for Cobas or HC2 is rather imprecise with only five BD-positive samples out of 333 tested. Nevertheless, 1.5% of discordant cases is in the range of what observed in other studies conducted in screening populations with clinically validated tests (16, 27). When comparing the positivity rates within our study, we observed 6.1% and 10.1% with Cobas and HC2, respectively. In comparison, the estimate of BD Onclarity was 6.8% and 8.2% if based on the sub-population tested with Cobas or on that tested with HC2. Among the cases positive for BD Onclarity and negative for the other two assays, HPV16 positivity was recorded among those negative for HC2. Regarding the cases BD-positive and Cobas-negative, although both assays use the same methodology, the PCR target region of Cobas is the HPV L1 gene, while for BD Onclarity, the amplified region spans the E6 and E7 oncogenes. Since during HPV genome integration, part of the L1 region may be lost, whereas E6/E7 gene expression remains present (34), it has been argued that a test searching for L1 expression may test falsely negative, but a recent study (35) has shown the similar performance of L1 and E6/E7 targeting assays for the detection of hrHPV types.

We found 751 Cobas or HC2 HPV-positive samples testing negative with BD Onclarity. The percentage of BD Onclarity-negative samples was higher in all the biomarker profiles linked to lower risk of immediate or future CIN3; 28.4% and 15% among the NILM and ASC-US/LSIL categories (while it was 4.9% among ASC-H+ samples), and 30.4% and 39.7% in the p16/ki67 and E6-E7 mRNA-negatives, respectively (9.1% and 6.6%, respectively, in positives). As expected, the proportion of BD Onclarity-negatives was very low among women with CIN2 or CIN3.

Real-time PCR assays are qualitative in intended use but provide CT values that are inversely correlated with the log amount of the targeted DNA in the specimen, thus giving an indication of the viral load. In Italy, the results of the HPV screening assays are reported as positive/negative, without any specification of CT values. To gain some insights into the distribution of the CT values, we analyzed the CT values for each channel of the Onclarity assay. We observed lower CT values in samples with positive results for cytology, p16/ki67, or E6/E7 mRNA. At the same time, no clear pattern was identified in their distribution in relation to the HPV-DNA test (HC2 or Cobas) that determined HPV positivity. These results are in line with the observation that the CT values on Cobas 4800 were directly correlated to the severity of cervical lesions (36), thus suggesting their possible role as risk indicators. Indeed, although lower CT values generally correspond to higher amounts of viral sequences (37), more data are needed since CT values can vary significantly between and within methods (38). Trying to increase sensitivity by increasing the CT thresholds decreases the overall accuracy of the test in a screening population. For the samples originally tested with HC2, unfortunately, we were unable to evaluate the agreement between the semiquantitative indications of viral load, i.e., HC2 ratio value, and BD Onclarity CT value, because this information was not available for all the samples tested with HC2.

The introduction of HPV genotyping as triage biomarker or risk indicator in routine population-based cervical cancer screening necessitates high-throughput and affordable assays. While partial HPV16/HPV18 genotyping selects cases with a higher risk of lesions and is already included in screening protocols, extended or complete genotyping must take into consideration the different carcinogenic risks of the other hrHPVs (14). Furthermore, partial HPV16/HPV18 genotyping, and to a lesser extent, also extended genotyping are already available as reflex results in some clinically validated assays. Analysis by type-grouping appears to be a reasonable strategy and is used in the BD Onclarity HPV assay. How to group the hrHPV types in a genotyping assay is not an easy task. While the BD Onclarity grouping does not completely reflect the specific risks and distribution of HPV types circulating in Italy (15–17), it must be acknowledged that a “perfect” grouping (good in all geographical areas) is not foreseeable. This is an important point to be considered when grouping different genotypes. In fact, the overall risk for cervical cancer of a group of genotypes may be affected by the prevalence of single HPV types, which varies according to the geographical region. For example, Del Mistro et al. (15) described that, in the Italian population of the NTCC study, HPV33 and 35 had a low prevalence but were among those with a high absolute risk of CIN3.

### Conclusion

Our study confirms some disagreement among different HPV assays used for screening, particularly in HPV-positive cases without lesions. We found a substantial agreement for women p16/ki67 or mRNA-positive at triage, with high-grade cytology and histologically confirmed CIN2 and CIN3, thus confirming a good clinical performance of all the tests used.

## Data Availability

Individual participant data that underlie the results reported in this article, after de-identification, are available for investigators whose proposed use of the data have been approved by the S. Giovanni Battista University Hospital Ethic committee, Turin, Italy. Proposals should be directed to paolo.giorgirossi@ausl.re.it and to comitatoetico@cittadellasalute.to.it. To gain access, data requestors will need to sign a data access agreement. The study protocol is freely available online.

## SUPPLEMENTAL MATERIAL

Supplemental material spectrum.00897-24-s0001.docx Figures S1 to S3; Tables S1 to S5.

ASM does not own the copyrights to Supplemental Material that may be linked to, or accessed through, an article. The authors have granted ASM a non-exclusive, world-wide license to publish the Supplemental Material files. Please contact the corresponding author directly for reuse.
