# Supplementary material for: Extended HPV genotyping by the BD Onclarity assay: concordance with screening HPV-DNA assays, triage biomarkers, and histopathology in women from the NTCC2 study
Source: Microbiol Spectr. 2024 Nov 22;13(1):e00897-24. doi: 10.1128/spectrum.00897-24 (PMC11723576; doi:10.1128/spectrum.00897-24)
Supplement: Supplemental material — Figures S1 to S3; Tables S1 to S5. [file spectrum.00897-24-s0001.docx]

**SUPPLEMENTARY MATERIAL**

**Supplementary Figure 1**. **Quantitative results based on cytology and BD Onclarity channel on baseline samples of Cobas/HC2 HPV DNA-positive women.**

The vertical red line indicates the manufacturer-assessed CT value for positivity: CT 38.4 for HPV16 and at CT 34.2 for the housekeeping gene and/or the other HPV types.

**Supplementary Figure 2**. **Quantitative results by p16/ki67 and BD Onclarity channel on baseline samples of Cobas/HC2 HPV DNA-positive women.**

The vertical red line indicates the manufacturer-assessed CT value for positivity: CT 38.4 for HPV16 and at CT 34.2 for the housekeeping gene and/or the other HPV types.

**Supplementary Figure 3. Quantitative results by mRNA and BD channel on baseline samples of Cobas/HC2 HPV DNA-positive women.**

The vertical red line indicates the manufacturer-assessed CT value for positivity: CT 38.4 for HPV16 and at CT 34.2 for the housekeeping gene and/or the other HPV types.

**Supplementary Table 1**. **Comparison of the HPV genotypes detected by the three hrHPV-DNA validated assays used, specified by test’s channel(s).**

| **HPV genotype** | **hrHPV assays** | |  |  |
| --- | --- | --- | --- | --- |
|  | **BD Onclarity** | **Cobas 4800** | | **HC2** |
| **16** | **Yes, alone** | **Yes, alone** | | **Yes** |
| **18** | **Yes, alone** | **Yes, alone** | | **Yes** |
| **31** | **Yes, alone** | **Yes, ch3** | | **Yes** |
| **33** | **Yes, ch p1** | **Yes, ch3** | | **Yes** |
| **35** | **Yes, ch p3** | **Yes, ch3** | | **Yes** |
| **39** | **Yes, ch p3** | **Yes, ch3** | | **Yes** |
| **45** | **Yes, alone** | **Yes, ch3** | | **Yes** |
| **51** | **Yes, alone** | **Yes, ch3** | | **Yes** |
| **52** | **Yes, alone** | **Yes, ch3** | | **Yes** |
| **56** | **Yes, ch p2** | **Yes, ch3** | | **Yes** |
| **58** | **Yes, ch p1** | **Yes, ch3** | | **Yes** |
| **59** | **Yes, ch p2** | **Yes, ch3** | | **Yes** |
| **66** | **Yes, ch p2** | **Yes, ch3** | | **No** |
| **68** | **Yes, ch p3** | **Yes, ch3** | | **Yes** |

**Supplementary Table 2**. **Agreement between original HPV-DNA test and BD Onclarity results for all sample channels of women recruited in the centers of Florence and Perugia**

|  |  | | **Any original HPV test** | | | | | | | | |  | |  |  |
| --- | --- | --- | --- | --- | --- | --- | --- | --- | --- | --- | --- | --- | --- | --- | --- |
|  |  | | **Positive** | | | | | **Negative** | | | **Total** | Agreement in the sample 82.2% | | | |
| **BD** **Onclarity** | **Positive** | | 1342 | | | | | 5 | | | 1347 | Cohen κ in the sample 0.54 (95% CI 0.50–0.59) | | | |
|  | **Negative** | | 356 | | | | | 327 | | | 683 | Agreement in the screening population* 97.1% | | | |
|  | **Total** | | 1698 | | | | | 332 | | | 2030 | Cohen κ in screening pop.* 0.78 (95% CI 0.77–0.80) | | | |
|  |  | | **HC2** | | | | | | | | |  | | | |
|  |  | | **Positive** | | | | **Negative** | | **Total** | | | Agreement in the sample 70.4% | | | |
| **BD Onclarity** | **Positive** | | 458 | | | | 3 | | 461 | | | Cohen κ in the sample 0.38 (95% CI 0.31–0.44) | | | |
|  | **Negative** | | 251 | | | | 146 | | 397 | | | Agreement in the screening population* 95.0% | | | |
|  | **Total** | | 709 | | | | 149 | | 858 | | | Cohen κ in screening pop.* 0.67 (95% CI 0.64–0.70) | | | |
|  |  | | **Cobas 4800** | | | | | | | | |  | | |  |
|  |  | | **Positive** | | **Negative** | | | | **Total** | | | Agreement in the sample 90.9% | | | |
| **BD Onclarity** | **Positive** | | 884 | | 2 | | | | 886 | | | Cohen κ in the sample 0.72 (95% CI 0.67–0.77) | | | |
|  | **Negative** | | 105 | | 181 | | | | 286 | | | Agreement in the screening population* 98.4% | | | |
|  | **Total** | | 989 | | 183 | | | | 1172 | | | Cohen κ in screening pop.* 0.85 (95% CI 0.84–0.87) | | | |
| **HPV16** |  | | **Cobas 4800** | | | | | | | | |  | | |  |
|  |  | | **Positive** | | **Negative** | | | | **Total** | | | Agreement in the sample 97.7% | | | |
| **BD Onclarity** | **Positive** | | 189 | | 15 | | | | 204 | | | Cohen κ in the sample 0.93 (95% CI 0.90–0.96) | | | |
|  | **Negative** | | 8 | | 777 | | | | 785 | | | Agreement in the screening population* 97.7% | | | |
|  | **Total** | | 197 | | 792 | | | | 989 | | | Cohen κ in screening pop.* 0.93 (95% CI 0.90–0.96) | | | |
| **HPV16/18** | |  | | **Cobas 4800** | | | | | | | | |  | |  |
|  | |  | | **Positive** | | **Negative** | | | | **Total** | | | Agreement in the sample 96.7% | | |
| **BD Onclarity** | | **Positive** | | 243 | | 17 | | | | 260 | | | Cohen κ in the sample 0.91 (95% CI 0.88–0.94) | | |
|  |  | **Negative** | | 16 | | 713 | | | | 728 | | | Agreement in the screening population* 96.7% | | |
|  |  | **Total** | | 259 | | 730 | | | | 989 | | | Cohen κ in screening pop.* 0.91 (95% CI 0.88–0.94) | | |

* The baseline samples negative for original HPV test are weighted to estimate the agreement and kappa values in a screening population with overall HPV DNA positivity of 7.8% (6.1% for Cobas 4800 and 10.1% for HC2)

**Supplementary Table 3**. **Agreement between original HPV-DNA Sample and BD Onclarity at the threshold of ≤40 CT for all channels of women recruited in the centers of Florence and Perugia (raw data).**

|  | |  | | | | **Any original HPV test** | | | | | | | | | | | |  |  |  |  |  |  |  |
| --- | --- | --- | --- | --- | --- | --- | --- | --- | --- | --- | --- | --- | --- | --- | --- | --- | --- | --- | --- | --- | --- | --- | --- | --- |
|  | | |  | | | | **Positive** | | | | | | **Negative** | | | | | **Total** | | | | | Agreement in the sample 89.6% | |
| **BD Onclarity** | | | **Positive** | | | | 1500 | | | | | | 14 | | | | | 1514 | | | | | Cohen κ in the sample 0.69 (95% CI 0.65–0.75) | |
|  |  |  | **Negative** | | | | 198 | | | | | | 318 | | | | | 516 | | | | | Agreement in the screening population 95.3% | |
|  |  |  | **Total** | | | | 1698 | | | | | | 332 | | | | | 2030 | | | | | Cohen κ in screening pop. 0.71 (95% CI 0.69–0.72) | |
|  | | |  | | | | **HC2** | | | | | | | | | | | | |  | | | | |
|  | | |  | | | | **Positive** | | | | **Negative** | | | | **Total** | | | | | Agreement in the sample 77.5% | | | | |
| **BD Onclarity** | | | **Positive** | | | | 525 | | | | 9 | | | | 534 | | | | | Cohen κ in the sample 0.46 (95% CI 0.40–0.53) | | | | |
|  |  |  | **Negative** | | | | 184 | | | | 140 | | | | 324 | | | | | Agreement in the screening population 92.2% | | | | |
|  |  |  | **Total** | | | | 709 | | | | 149 | | | | 858 | | | | | Cohen κ in screening pop. 0.59 (95% CI 0.56–0.62) | | | | |
|  | | |  | | | | **Cobas 4800** | | | | | | | | | | | | | |  | |  |  |
|  | | |  | | | | **Positive** | | | | **Negative** | | | | | **Total** | | | | | Agreement in the sample 98.4% | | | |
| **BD Onclarity** | | | **Positive** | | | | 975 | | | | 5 | | | | | 980 | | | | | Cohen κ in the sample 0.94 (95% CI 0.91–0.97) | | | |
|  |  |  | **Negative** | | | | 14 | | | | 178 | | | | | 192 | | | | | Agreement in the screening population 97.3% | | | |
|  |  |  | **Total** | | | | 989 | | | | 183 | | | | | 1172 | | | | | Cohen κ in screening pop. 0.80 (95% CI 0.78–0.82) | | | |
| **HPV16** | | |  | | | | **Cobas 4800** | | | | | | | | | | |  | | | | |  |  |
|  | | |  | | | | **Positive** | | **Negative** | | | | **Total** | | | | | Agreement in the sample 97.7% | | | | | | |
| **BD Onclarity** | | | **Positive** | | | | 190 | | 16 | | | | 206 | | | | | Cohen κ in the sample 0.93 (95% CI 0.90–0.96) | | | | | | |
|  |  |  | **Negative** | | | | 7 | | 776 | | | | 783 | | | | | Agreement in the screening population* 97.7% | | | | | | |
|  |  |  | **Total** | | | | 197 | | 792 | | | | 989 | | | | | Cohen κ in screening pop.* 0.93 (95% CI 0.90–0.96) | | | | | | |
| **HPV16/18** | | | |  | | | | **Cobas 4800** | | | | | | | | | | |  | | | |  |  |
|  | | | |  | | | | **Positive** | | **Negative** | | | | **Total** | | | | | Agreement in the sample 97.2% | | | | | |
| **BD Onclarity** | | | | **Positive** | | | | 249 | | 18 | | | | 267 | | | | | Cohen κ in the sample 0.93 (95% CI 0.90–0.95) | | | | | |
|  |  |  |  | **Negative** | | | | 10 | | 712 | | | | 722 | | | | | Agreement in the screening population* 97.2% | | | | | |
|  |  |  |  | **Total** | | | | 259 | | 730 | | | | 989 | | | | | Cohen κ in screening pop.* 0.93 (95% CI 0.90–0.95) | | | | | |

* The baseline samples negative for original HPV test are weighted to estimate the agreement and kappa values in a screening population with overall HPV DNA positivity of 7.8% (6.1% for Cobas 4800 and 10.1% for HC2)

**Supplementary Table 4. BD Onclarity typing result distribution according to women’s age (<35, 35-50, >50 years) at enrollment. (A) Samples tested by Cobas 4800. (B) Samples tested by HC2.**

**(A)**

| **BD Onclarity type** | **<35** | | **35-50** | | **>50** | |
| --- | --- | --- | --- | --- | --- | --- |
|  | **n** | **%** | **n** | **%** | **n** | **%** |
| Samples analyzed | 139 |  | 849 |  | 448 |  |
| 16 | 34 | 24.5 | 185 | 21.8 | 72 | 16.1 |
| 18 | 4 | 2.9 | 60 | 7.1 | 27 | 6.0 |
| 45 | 4 | 2.9 | 42 | 4.9 | 15 | 3.3 |
| 33/58 | 7 | 5.0 | 104 | 12.2 | 62 | 13.8 |
| 31 | 24 | 17.3 | 137 | 16.1 | 66 | 14.7 |
| 56/59/66 | 31 | 22.3 | 178 | 21.0 | 131 | 29.2 |
| 51 | 13 | 9.4 | 70 | 8.2 | 30 | 6.7 |
| 52 | 18 | 12.9 | 70 | 8.2 | 40 | 8.9 |
| 35/39/68 | 14 | 10.1 | 103 | 12.1 | 60 | 13.4 |
| Negative | 24 | 17.3 | 104 | 12.2 | 58 | 12.9 |
| At least 1 channel positives | 115 | 82.7 | 745 | 87.8 | 390 | 87.1 |
| Multichannel positives | 27 | 19.4 | 115 | 13.5 | 96 | 21.4 |

**(B)**

| **BD Onclarity type** | **<35** | | **35-50** | | **>50** | |
| --- | --- | --- | --- | --- | --- | --- |
|  | **n** | **%** | **n** | **%** | **n** | **%** |
| Samples analyzed | 213 |  | 1,044 |  | 436 |  |
| 16 | 36 | 16.9 | 152 | 14.6 | 53 | 12.2 |
| 18 | 9 | 4.2 | 33 | 3.2 | 18 | 4.1 |
| 45 | 3 | 1.4 | 40 | 3.8 | 19 | 4.4 |
| 33/58 | 22 | 10.3 | 79 | 7.6 | 43 | 9.9 |
| 31 | 24 | 11.3 | 122 | 11.7 | 41 | 9.4 |
| 56/59/66 | 40 | 18.8 | 153 | 14.7 | 84 | 19.3 |
| 51 | 24 | 11.3 | 66 | 6.3 | 17 | 3.9 |
| 52 | 16 | 7.5 | 71 | 6.8 | 22 | 5.0 |
| 35/39/68 | 25 | 11.7 | 117 | 11.2 | 52 | 11.9 |
| Negative | 58 | 27.2 | 363 | 34.8 | 157 | 36.0 |
| At least 1 channel positives | 155 | 72.8 | 681 | 65.2 | 279 | 64.0 |
| Multichannel positives | 36 | 16.9 | 128 | 12.3 | 57 | 13.1 |

**Supplementary Table 5. BD Onclarity typing result distribution according to the time (<5, >5 years) elapsed from original testing to genotyping. (A) Samples tested by Cobas 4800. (B) Samples tested by HC2.**

**(A)**

| **BD Onclarity type** | **<5 years** | | **>5 years** | |
| --- | --- | --- | --- | --- |
|  | **n** | **%** | **n** | **%** |
| Samples analyzed | 153 |  | 1,283 |  |
| 16 | 38 | 24.8 | 253 | 19.7 |
| 18 | 10 | 6.5 | 81 | 6.3 |
| 45 | 8 | 5.2 | 53 | 4.1 |
| 33/58 | 18 | 11.8 | 155 | 12.1 |
| 31 | 24 | 15.7 | 203 | 15.8 |
| 56/59/66 | 39 | 25.5 | 301 | 23.5 |
| 51 | 10 | 6.5 | 103 | 8.0 |
| 52 | 15 | 9.8 | 113 | 8.8 |
| 35/39/68 | 17 | 11.1 | 160 | 12.5 |
| Negative | 22 | 14.4 | 164 | 12.8 |
| At least 1 channel positives | 131 | 85.6 | 1,119 | 87.2 |
| Multichannel positives | 34 | 22.2 | 244 | 19.0 |

**(B)**

| **BD Onclarity type** | **<5 years** | | **>5 years** | |
| --- | --- | --- | --- | --- |
|  | **n** | **%** | **n** | **%** |
| Samples analyzed | 1,151 |  | 542 |  |
| 16 | 164 | 14.2 | 77 | 14.2 |
| 18 | 43 | 3.7 | 17 | 3.1 |
| 45 | 40 | 3.5 | 22 | 4.1 |
| 33/58 | 95 | 8.3 | 49 | 9.0 |
| 31 | 126 | 10.9 | 61 | 11.3 |
| 56/59/66 | 186 | 16.2 | 91 | 16.8 |
| 51 | 66 | 5.7 | 41 | 7.6 |
| 52 | 72 | 6.3 | 37 | 6.8 |
| 35/39/68 | 128 | 11.1 | 66 | 12.2 |
| Negative | 408 | 35.4 | 170 | 31.4 |
| At least 1 channel positives | 743 | 64.6 | 372 | 68.6 |
| Multichannel positives | 146 | 12.7 | 75 | 13.8 |
